# Effectiveness of Prehabilitation Programs in Reducing the Length of Hospital Stay and Complication Rates in Major Surgeries: A Systematic Review

**DOI:** 10.7759/cureus.76932

**Published:** 2025-01-05

**Authors:** Ebrahem H Mohamed, Sibthein A Khalid, Ehsan Ul Haq, Ismail S Abougendy, Sami Qadeer

**Affiliations:** 1 Surgery, Ministry of Health Holdings, Cairo, EGY; 2 Orthopaedics and Traumatology, Medical University of Plovdiv, Plovdiv, BGR; 3 Surgery, Pak International Medical College, Peshawar, PAK; 4 Surgery, Misr University for Science and Technology, Cairo, EGY; 5 Internal Medicine, Nishtar Medical University, Multan, PAK

**Keywords:** colorectal surgery, enhanced recovery pathways, functional recovery, postoperative complications, prehabilitation, randomized controlled trials

## Abstract

Prehabilitation, involving physical, nutritional, and psychological interventions before surgery, has emerged as a promising strategy to improve postoperative outcomes. This systematic review evaluated the impact of prehabilitation programs across various surgical systems, including cardiac, abdominal, colorectal, and thoracic surgeries. Data were synthesized from meta-analyses and systematic reviews to assess the effectiveness of uni- and multimodal prehabilitation interventions. Findings demonstrate that multimodal prehabilitation, particularly combining exercise and nutrition, significantly reduces postoperative complications, enhances functional recovery, and shortens hospital length of stay (LoS). While benefits were observed across surgical systems, variability in outcomes was noted due to differences in intervention design and patient populations. The review highlights the adaptability of prehabilitation and underscores its potential as a cost-effective and scalable approach to optimize surgical outcomes. However, gaps remain in standardizing protocols and evaluating long-term benefits, particularly for underrepresented surgeries such as spine and thoracic procedures. These findings support the integration of tailored prehabilitation programs into perioperative care and emphasize the need for further high-quality research.

## Introduction and background

Prehabilitation, a proactive strategy to enhance patient outcomes before major surgery, has gained significant recognition in recent years [1,2]. It involves interventions such as physical exercise, nutritional optimization, and psychological support to strengthen a patient's physiological and functional reserve [3]. By targeting modifiable risk factors, prehabilitation aims to reduce postoperative complications, shorten hospital stays, and improve recovery, particularly in high-stress major surgeries [1]. Despite the increasing evidence supporting prehabilitation, its effectiveness across various surgical systems remains under investigation.

Research suggests that prehabilitation can be tailored to specific surgical needs, including cardiac, abdominal, and oncologic procedures [4]. However, inconsistencies in intervention design, patient demographics, and outcome measures complicate the generalization of findings. This systematic review evaluates the impact of prehabilitation on reducing hospital length of stay (LoS) and postoperative complications, synthesizing evidence across diverse surgical systems [5].

Using the population, intervention, control, and outcomes (PICO) framework, this review systematically examines the population (P) of patients undergoing major elective surgeries, such as cardiac, abdominal, thoracic, and colorectal procedures. The intervention (I) includes uni- or multimodal prehabilitation strategies, such as exercise, nutritional support, and psychological preparation. The control (C) is standard preoperative care without prehabilitation, and the outcomes (O) focus on hospital LoS, postoperative complications, and overall functional recovery [6]. This structured approach provides actionable insights into integrating prehabilitation into perioperative care for optimal patient outcomes.

## Review

Materials and methods

Search Strategy

The search strategy for this systematic review was designed to ensure a comprehensive and unbiased identification of relevant studies. Multiple electronic databases, including PubMed, Excerpta Medica database (Embase), Cochrane Library, Cumulative Index to Nursing and Allied Health Literature (CINAHL), and Scopus, were systematically searched for peer-reviewed articles. The search terms were carefully developed and combined using Boolean operators to capture studies related to prehabilitation across various surgical systems, with key concepts including "prehabilitation," "surgery," "postoperative outcomes," and "multimodal interventions." Additional searches were conducted in gray literature sources to minimize publication bias. No language restrictions were applied, and reference lists of included studies were manually screened for additional eligible articles. The Preferred Reporting Items for Systematic Reviews and Meta-Analyses (PRISMA) guidelines [7] were followed to ensure transparency and reproducibility in study selection, screening, and inclusion. This rigorous approach provided a robust foundation for synthesizing high-quality evidence for the review.

Eligibility Criteria

The eligibility criteria for this systematic review were established to identify studies evaluating the impact of prehabilitation on postoperative outcomes across various surgical systems [8]. Inclusion criteria encompassed randomized controlled trials (RCTs) and meta-analyses published in peer-reviewed journals, focusing on adult patients (≥18 years) undergoing elective surgeries, including cardiac, abdominal, colorectal, and thoracic procedures. Studies that investigated uni- or multimodal prehabilitation interventions, such as exercise-based programs, nutritional support, and psychological preparation, were included. Outcomes of interest comprised hospital LoS, postoperative complications, functional recovery, and patient-reported measures like quality of life. Exclusion criteria included studies without a control group, those focusing on emergency surgeries, or interventions unrelated to prehabilitation. Articles with incomplete data or duplicate publications were also excluded. This rigorous criterion ensured the inclusion of high-quality, relevant studies to address the research question comprehensively.

Data Extraction

Data extraction for this systematic review was performed systematically to ensure accuracy and comprehensiveness. Two independent reviewers extracted data from each included study using a standardized data extraction form. Key variables collected included study characteristics (authors, year, and study design), population demographics (age, type of surgery, and sample size), intervention details (type, duration, and modality of prehabilitation), comparator interventions, and reported outcomes (e.g., hospital LoS, postoperative complications, functional recovery metrics). Statistical details, such as effect sizes, confidence intervals, and measures of heterogeneity, were also extracted to facilitate meta-analysis. Any discrepancies between the reviewers were resolved through discussion or consultation with a third reviewer to maintain consistency and reduce bias. The extracted data were organized in a tabular format for synthesis, adhering to PRISMA guidelines to ensure transparency and reproducibility throughout the review process.

Data Analysis and Synthesis

Data analysis and synthesis for this systematic review were conducted qualitatively to provide a comprehensive and contextual evaluation of the included meta-analyses. Key findings from each study were extracted and summarized to identify trends, patterns, and gaps in the evidence regarding the impact of prehabilitation on postoperative outcomes across various surgical systems. The results were synthesized narratively, emphasizing comparisons of intervention types (e.g., multimodal vs. unimodal), surgical specialties, and reported outcomes such as hospital LoS, postoperative complications, and functional recovery. The synthesis aimed to highlight areas of agreement and divergence among the included meta-analyses and their implications for clinical practice. Heterogeneity in study designs, populations, and interventions was discussed qualitatively to provide context for the variability in findings. This approach ensured a rigorous and systematic evaluation of the existing literature without conducting a new meta-analysis, aligning with the review's objectives.

Results

Study Selection Process

**Figure 1:**
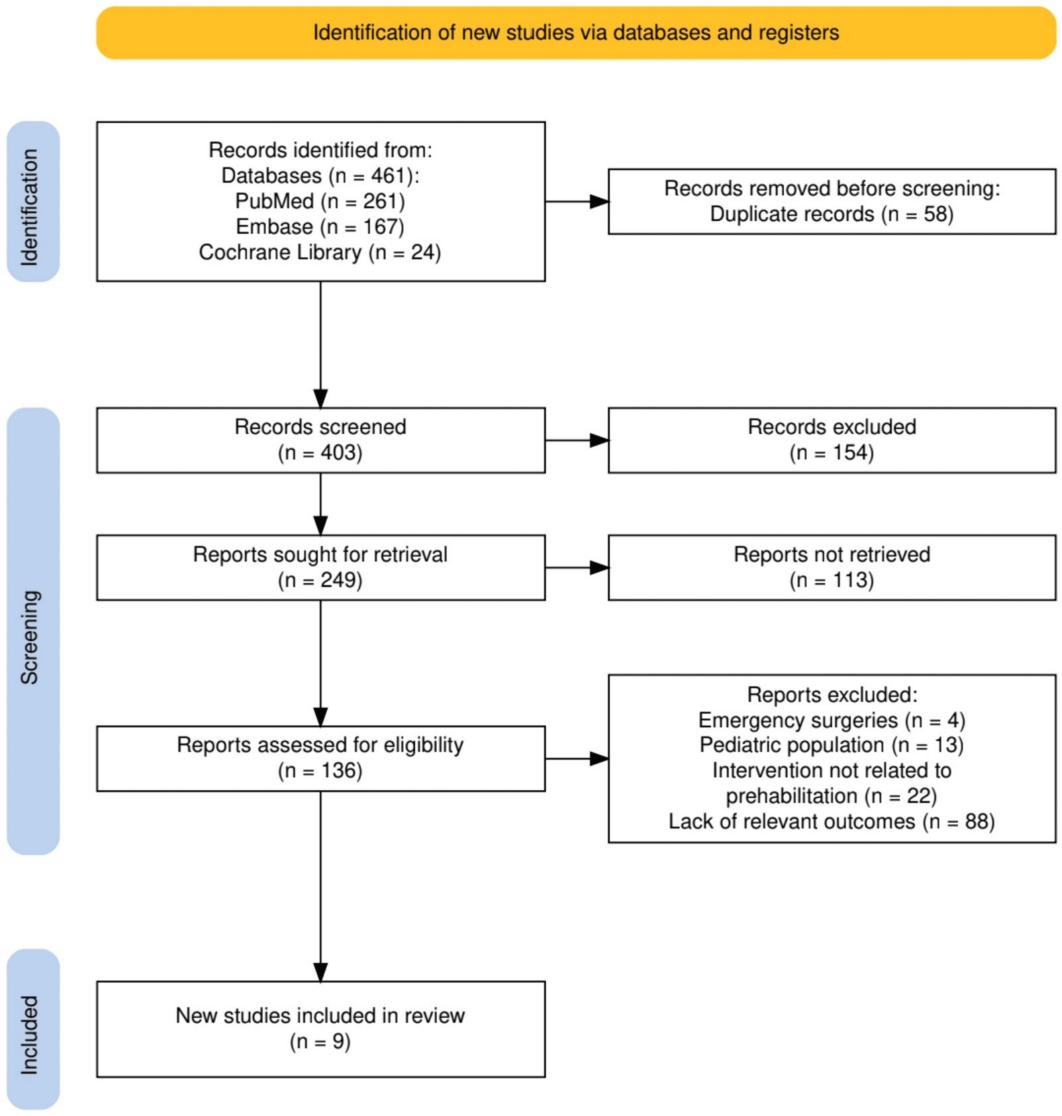
The PRISMA flowchart represents the study selection process PRISMA: Preferred Reporting Items for Systematic Reviews and Meta-Analyses; Embase: Excerpta Medica database

The study selection process followed PRISMA guidelines and is outlined in Figure 1. Initially, 461 records were identified through database searches in PubMed (n = 261), Embase (n = 167), and Cochrane Library (n = 24). After removing 58 duplicate records, 403 studies were screened based on titles and abstracts, with 154 excluded for not meeting inclusion criteria. From 249 reports sought for retrieval, 113 could not be retrieved. Subsequently, 136 full-text articles were assessed for eligibility, leading to the exclusion of studies due to reasons such as emergency surgeries (n = 4), pediatric populations (n = 13), interventions not related to prehabilitation (n = 22), and lack of relevant outcomes (n = 88). Ultimately, nine studies were included in the systematic review for qualitative synthesis. This rigorous selection ensured the inclusion of high-quality and relevant studies.

Characteristics of the Selected Studies

The study selection process involved a systematic approach to identify relevant meta-analyses and systematic reviews examining the impact of prehabilitation on postoperative outcomes across various surgical systems. As detailed in Table 1, studies were included if they evaluated uni- or multimodal prehabilitation interventions in adult surgical populations, focusing on outcomes such as hospital LoS, postoperative complications, and functional recovery. After initial screening, only studies meeting stringent inclusion criteria, such as randomized controlled trials or meta-analyses with robust methodologies, were selected. The final selection included diverse surgical specialties, such as cardiac, abdominal, colorectal, and upper abdominal surgeries, ensuring the broad applicability of findings. This rigorous selection process resulted in the inclusion of high-quality studies that collectively provided comprehensive insights into the efficacy of prehabilitation interventions.

**Table 1:** A summary of the characteristics of the selected studies IMT: inspiratory muscle training; LoS: length of stay; CI: confidence interval; RR: risk ratio; RCTs: randomized controlled trials; SOC: standard of care; ICU: intensive care unit; OR: odds ratio; MD: mean difference; AF: atrial fibrillation; AE: aerobic exercise; WBC: white blood cell count; PPCs: postoperative pulmonary complications; Log-OR: log-odds ratio

Authors (Year)	Population	Intervention	Comparison	Outcomes	Statistical data	Key findings
Perry et al. (2021) [5]	Patients (≥18 years) undergoing major elective surgery (curative or palliative)	IMT, immunonutrition, multimodal interventions, exercise, and smoking cessation were administered in the preoperative period	Standard care without prehabilitation	Reduced hospital LoS, fewer postoperative pulmonary and infective complications, reduced wound infections, improved pulmonary outcomes	IMT LoS: -1.81 days (95% CI -2.31 to -1.31); Immunonutrition LoS: -2.11 days (95% CI -3.07 to -1.15); Pulmonary complications: IMT RR 0.55 (95% CI 0.38–0.80), Exercise RR 0.54 (95% CI 0.39–0.75)	Multimodal prehabilitation interventions, including IMT and immunonutrition, reduced LoS and complications. Smoking cessation reduced wound infections (RR 0.28, 95% CI 0.12–0.64). The overall evidence quality was low.
Cambriel et al. (2023) [9]	Patients undergoing elective surgery (2090 patients randomized 1:1 in 25 RCTs)	Prehabilitation programs lasting ≥14 days (uni- or multimodal) targeting preoperative health improvement	SOC	ICU LoS, hospital LoS, postoperative complications, and reported pain at postoperative day 1	ICU LoS: -0.57 days (95% CI -1.10 to -0.04, P = 0.03, I² = 46%); Complication rates: OR 1.02 (95% CI 0.93–1.13, P = 0.10, I² = 34%); Hospital LoS: -0.13 days (95% CI -0.56 to 0.28, P = 0.53, I² = 21%)	Prehabilitation significantly reduced ICU LoS compared with SOC but had no effect on postoperative complications, hospital LoS, or pain scores. Programs require standardization and patient-specific targeting.
Ricci et al. (2024) [10]	Patients undergoing major abdominal surgery (25 studies included)	Physical prehabilitation (AE, IMT, or resistance training)	Standard care without prehabilitation	Reduced overall morbidity, pneumonia, and LoS; no significant impact on mortality	Overall morbidity: AE OR 0.61, P-score = 0.76; AE + IMT OR 0.66, P-score = 0.68; Pneumonia: AE + IMT OR 0.21, P-score = 0.91; LoS: AE MD -1.63 days (95% CI -3.43 to 0.18), AE + IMT MD -1.70 days (95% CI -2.06 to -1.27)	AE combined with IMT had the most significant impact on reducing morbidity, pneumonia, and LoS. AE alone was effective but less so than the combination of AE + IMT.
Steinmetz et al. (2023) [11]	Adults scheduled for elective cardiac surgery (six studies, n = 665)	Exercise-based prehabilitation	Standard care without prehabilitation	Improved six-min walking distance (pre- and post-surgery), reduced LoS, lower risk of postoperative AF	Six-min walking distance: post-intervention MD 75.4 m (95% CI 13.7–137.1, P = 0.02), Post-surgery MD 30.5 m (95% CI 8.5–52.6, P = 0.007); LoS: MD -1.00 day (95% CI -1.78 to -0.23, P = 0.01); AF: RR 0.34 (95% CI 0.14–0.83, P = 0.02)	Exercise-based prehabilitation significantly improved functional capacity, reduced LoS, and decreased the risk of postoperative AF in patients ≤65 years.
Shen et al. (2024) [12]	Adults with esophagogastric cancer undergoing esophagectomy or gastrectomy (661 patients; eight studies: five RCTs and three cohort studies)	Nutrition-based prehabilitation (uni- or multimodal, ≥1 week)	Standard care or unimodal prehabilitation	Reduced risk of postoperative complications, decreased LoS	Postoperative complications: RR 0.78 (95% CI 0.66–0.93); Any prehabilitation: RR 0.77 (95% CI 0.66–0.90); LoS: MD -0.77 days (95% CI -1.46 to -0.09)	Multimodal nutrition-based prehabilitation reduced postoperative complications significantly and slightly decreased LOS. Unimodal prehabilitation showed no significant effect. Additional rigorous RCTs are needed.
Zhou et al. (2024) [13]	Patients undergoing colorectal surgery (17 RCTs, n = 1961)	Multimodal prehabilitation (including exercise, nutrition, and psychological support) and exercise prehabilitation	Standard care or exercise prehabilitation	Improved functional capacity (six-min walk test) with multimodal prehabilitation but no effect on LoS, complications, anxiety, or depression scores	Functional capacity: Multimodal prehabilitation MD 29.00 (95% CI 26.64–31.36); Preoperative subgroup: MD 34.77 (95% CI 16.76–52.77); Exercise prehabilitation showed no significant effects on any outcomes	Multimodal prehabilitation improved preoperative functional capacity but had no effect on LoS, postoperative complications, or anxiety and depression. Exercise prehabilitation showed no significant benefits.
Franssen et al. (2024) [14]	Patients with colorectal cancer undergoing surgery (RCTs and observational studies)	Prehabilitation programs before colorectal surgery	Standard care or no prehabilitation	Reduction in postoperative complications and LoS in observational studies, but no significant effect in RCTs	Observational studies: Complications OR 0.54 (95% CI 0.40–0.72), LoS MD -1.34 days (95% CI -2.57 to -0.12); RCTs: Complications OR 0.95 (95% CI 0.53–1.72), LoS MD 0.16 days (95% CI -0.52 to 0.83)	Observational studies suggest significant reductions in complications and LoS, reflecting real-life practice. RCTs did not show significant benefits, highlighting the need for real-life-focused studies.
Zheng & Zhang (2020) [15]	Patients undergoing cardiac surgery (six studies included)	Preoperative exercise (inspiratory muscle training, aerobics, resistance training, and stretching)	Standard care or no prehabilitation	Reduced length of ICU stay and improved physical function; no significant difference in WBC count or mental health after surgery	ICU stay: MD -1.35 days (95% CI -2.64 to -0.06, P = 0.04, I² = 88%); Physical function: Z = 9.92, P < 0.00001; WBC count: No significant difference	Preoperative exercise improved ICU recovery time and postoperative physical function but did not affect WBC count or mental health outcomes. Limited publication bias was observed.
Amirkhosravi et al. (2024) [16]	Patients undergoing elective upper abdominal surgery (10 studies, n = 1503)	Multimodal prehabilitation (including exercise, nutrition, and psychological support)	Standard care or no prehabilitation	Reduced PPCs and all-cause complications; no significant reduction in hospital LoS unless exercise was included	All-cause complications: Log-OR -0.38 (95% CI -0.75 to -0.004, P = 0.048); PPCs: Log-OR -0.96 (95% CI -1.38 to -0.54, P < 0.001); LoS with exercise: MD -0.91 days (95% CI -1.67 to -0.14, P = 0.02)	Multimodal prehabilitation significantly reduced PPCs and overall complications in upper abdominal surgeries. Exercise-based programs reduced hospital LoS, but multimodal interventions without exercise did not.

Quality Assessment

The quality assessment of the included meta-analyses, summarized in Table 2, reflects a combination of high- and moderate-quality studies based on rigorous evaluation criteria. Studies rated as high quality demonstrated robust methodologies, including clearly defined research questions, protocol registration, comprehensive search strategies, thorough risk-of-bias assessments, appropriate statistical methods, and effective evaluation of publication bias and heterogeneity. Moderate-quality studies, while maintaining strong methodological frameworks in terms of statistical and bias assessments, lacked certain elements such as protocol registration or adequate consideration of publication bias. Despite these limitations, all included studies provided valuable insights into the effectiveness of prehabilitation interventions, ensuring a balanced and comprehensive synthesis of the evidence. The quality assessment underscores the importance of standardizing methodological practices, such as protocol registration and publication bias consideration, to enhance the reliability and applicability of future research.

**Table 2:** A summary of the quality assessment of each included study PROSPERO: International Prospective Register of Systematic Reviews; GRADE: grading of recommendations assessment, development, and evaluation; CINeMA: confidence in network meta-analysis; RCT: RCT: randomized controlled trial

Authors (year)	Research question clearly defined	Protocol registered (e.g., PROSPERO)	Comprehensive search strategy	Risk of bias assessed	Appropriate statistical methods	Publication bias considered	Heterogeneity assessed	Overall quality rating	Comments
Perry et al. (2021) [5]	Yes	Yes	Yes	Yes	Yes	Yes	Yes	High	Well-conducted meta-analysis with strong methodology, comprehensive risk-of-bias assessment, and heterogeneity analysis.
Cambriel et al. (2023) [9]	Yes	No	Yes	Yes	Yes	No	Yes	Moderate	Lacked protocol registration; publication bias not adequately addressed. Otherwise, robust methods used.
Ricci et al. (2024) [10]	Yes	Yes	Yes	Yes	Yes	Yes	Yes	High	Comprehensive component network meta-analysis using GRADE/CINeMA with robust quality assessment and heterogeneity evaluation.
Steinmetz et al. (2023) [11]	Yes	No	Yes	Yes	Yes	No	Yes	Moderate	Did not register a protocol or consider publication bias, but statistical methods and risk of bias assessment were robust.
Shen et al. (2024) [12]	Yes	Yes	Yes	Yes	Yes	Yes	Yes	High	High-quality meta-analysis with clear methodology, robust risk-of-bias assessment, and statistical analysis.
Zhou et al. (2024)[13]	Yes	No	Yes	Yes	Yes	No	Yes	Moderate	No protocol registration or publication bias assessment; comprehensive heterogeneity analysis and risk of bias assessment conducted.
Franssen et al. (2024) [14]	Yes	No	Yes	Yes	Yes	No	Yes	Moderate	Protocol registration missing; publication bias not considered. Balanced comparison between RCTs and observational studies.
Zheng & Zhang (2020) [15]	Yes	No	Yes	Yes	Yes	No	Yes	Moderate	No protocol registration or publication bias consideration. Strong heterogeneity and bias evaluation.
Amirkhosravi et al. (2024) [16]	Yes	Yes	Yes	Yes	Yes	Yes	Yes	High	Comprehensive methods and statistical approaches; heterogeneity and publication bias thoroughly assessed.

Prehabilitation Programs

To provide a comprehensive overview of the effectiveness of various prehabilitation programs across different surgical specialties, a summary of the key findings from the reviewed studies is presented in Table 3. This table outlines the specific types of surgeries, the corresponding prehabilitation interventions, the level of evidence supporting each program, and the key postoperative outcomes observed. The prehabilitation interventions include uni- and multimodal approaches, such as inspiratory muscle training (IMT), aerobic exercise (AE), nutritional support, and psychological interventions, tailored to meet the unique needs of patients undergoing major surgeries. The levels of evidence are categorized based on RCTs, meta-analyses, and observational studies, ensuring the reliability of the reported findings. The table demonstrates that multimodal prehabilitation programs, particularly those combining exercise and nutritional support, consistently show better outcomes in terms of reduced LoS, fewer postoperative complications, and improved functional recovery. However, variability in results across different surgical specialties highlights the need for further standardization of prehabilitation protocols. Overall, Table 3 serves as a practical guide for clinicians to tailor prehabilitation strategies according to the specific needs of surgical patients, based on the best available evidence.

**Table 3:** A summary of the prehabilitation programs IMT: inspiratory muscle training; LoS: length of stay; ICU: intensive care unit; AE: aerobic exercise; RCTs: randomized controlled trials; WBC: white blood cell

Surgery type	Prehabilitation program	Level of evidence	Key outcomes
Major elective surgery	IMT, immunonutrition, multimodal interventions, exercise, smoking cessation	Moderate (meta-analysis)	Reduced LoS, fewer complications, reduced wound infections, improved pulmonary outcomes
Elective surgery	Prehabilitation programs lasting ≥14 days (uni- or multimodal)	Moderate (RCTs)	Reduced ICU LoS, no significant effect on complications or hospital LoS
Major abdominal surgery	Physical prehabilitation (AE, IMT, or resistance training)	High (meta-analysis)	Reduced morbidity, pneumonia, LoS; no impact on mortality
Cardiac surgery	Exercise-based prehabilitation	High (RCTs)	Improved functional capacity, reduced LoS, decreased risk of atrial fibrillation
Esophagogastric cancer surgery	Nutrition-based prehabilitation (uni- or multimodal, ≥1 week)	Moderate (RCTs and cohort studies)	Reduced complications, decreased LoS
Colorectal surgery	Multimodal prehabilitation (exercise, nutrition, psychological support) and exercise prehabilitation	High (RCTs)	Improved functional capacity, no effect on LoS, complications, anxiety, or depression
Colorectal cancer surgery	Prehabilitation programs before colorectal surgery	Moderate (RCTs and observational studies)	Reduced complications and LoS in observational studies; no significant effect in RCTs
Cardiac surgery	Preoperative exercise (IMT, aerobics, resistance training, stretching)	Moderate (RCTs)	Reduced ICU stay, improved physical function, no significant difference in WBC count or mental health
Upper abdominal surgery	Multimodal prehabilitation (exercise, nutrition, psychological support)	Moderate (RCTs)	Reduced pulmonary complications, reduced all-cause complications, no significant reduction in LoS unless exercise included

Discussion

This systematic review synthesizes evidence on the effectiveness of prehabilitation programs across various surgical systems, highlighting their potential to improve postoperative outcomes. The review demonstrates that multimodal prehabilitation approaches, combining exercise, nutrition, and psychological support, consistently yield significant benefits across different patient populations. For example, Perry et al. [5] reported that IMT and immunonutrition significantly reduced hospital LoS and pulmonary complications. Similarly, Ricci et al. [10] demonstrated that the combination of AE with IMT was particularly effective in reducing morbidity, pneumonia rates, and LoS. These findings emphasize the critical role of tailored, integrated prehabilitation protocols in enhancing recovery and reducing complications, particularly in high-risk surgical patients.

Evidence from exercise-based interventions specifically supports improved functional capacity and postoperative recovery in cardiac and colorectal surgeries. Steinmetz et al. showed that exercise-based prehabilitation significantly enhanced patients' six-minute walking distance and reduced hospital LoS following elective cardiac surgery. In colorectal surgery, Zhou et al. [13] found that multimodal prehabilitation improved preoperative functional capacity but had a limited impact on hospital LoS and complication rates. Overall, observational studies, such as those by Franssen et al. [14], demonstrated real-world reductions in complications and LoS with prehabilitation strategies, underscoring their practical applicability. However, the variability in results across surgical types and intervention modalities highlights the importance of tailoring prehabilitation programs to specific surgical contexts to achieve optimal outcomes.

The impact of prehabilitation varies significantly across surgical specialties due to the unique physiological demands of each procedure. Exercise-based interventions show substantial benefits in cardiac surgeries, as demonstrated by Steinmetz et al. [11], while abdominal surgeries achieve superior outcomes through multimodal approaches that integrate inspiratory muscle training and nutritional optimization [10]. Prehabilitation in colorectal surgeries enhances functional capacity, but its effect on reducing complications and hospital stays remains limited [13]. Spine surgeries, which are underrepresented in the existing literature, present a promising area for prehabilitation research. Tailored exercise and nutrition programs could potentially address musculoskeletal impairments, improving recovery outcomes in these patients [17,18]. The adaptability of prehabilitation across diverse surgical systems highlights the need for future investigations to determine its role in less-studied procedures [19].

This review aligns with previous research on the benefits of multimodal prehabilitation in improving postoperative outcomes [20]. Prior studies consistently report reductions in pulmonary complications and enhancements in functional capacity with combined exercise and nutrition interventions. Our findings corroborate these trends, with evidence from Perry et al. [5] and Ricci et al. [10] showing significant reductions in hospital LoS and complication rates. However, studies like Cambriel et al. [9] reveal variability in prehabilitation effectiveness, particularly across different surgical types and patient populations. By explicitly comparing unimodal and multimodal interventions, this review broadens existing knowledge and provides novel insights into the superior efficacy of combined approaches. The findings emphasize the importance of developing standardized, patient-specific protocols to maximize the benefits of prehabilitation in perioperative care [21].

This systematic review demonstrates key methodological strengths, including a comprehensive search strategy across multiple databases, adherence to PRISMA guidelines, and the inclusion of diverse surgical systems, thereby enhancing the generalizability of its findings. By synthesizing data from both RCTs and observational studies within existing meta-analyses, the review provides valuable insights into prehabilitation's effectiveness across controlled and real-world settings. However, moderate heterogeneity in prehabilitation protocols, variability in study quality, and the limited number of RCTs for certain surgical specialties, such as thoracic and oncologic procedures, remain significant limitations. These factors hinder the ability to draw definitive conclusions. Furthermore, potential biases in the included meta-analyses, despite efforts to address them, highlight the ongoing need for standardized prehabilitation protocols and further high-quality research to strengthen the evidence base and ensure consistency in clinical practice.

The findings of this systematic review underscore the practical benefits of integrating prehabilitation into routine perioperative care. Multimodal prehabilitation programs that combine exercise, nutrition, and psychological support have consistently demonstrated reductions in postoperative complications and hospital LoS, positioning them as cost-effective strategies to enhance surgical outcomes [22]. For instance, interventions such as IMT and aerobic exercise, when combined with nutritional support, significantly improve recovery metrics, as highlighted by Ricci et al. [10] and Perry et al. [5]. The adaptability of these interventions across various surgical systems and patient populations further supports their feasibility. Tailored prehabilitation protocols can be implemented through hospital-based programs or home-based models, with remote monitoring technologies facilitating broader access [23]. By enhancing functional capacity and reducing complications, prehabilitation programs not only improve patient outcomes but also alleviate healthcare resource burdens, making them a valuable addition to perioperative care pathways. Healthcare institutions, particularly in resource-limited settings, should prioritize prehabilitation as part of surgical planning for high-risk patients.

Future research should aim to address the gaps and limitations identified in this review, particularly the need for standardized prehabilitation protocols that are specific to different surgical systems and patient populations [24]. Large-scale, high-quality RCTs are essential to validate findings across underrepresented procedures, such as thoracic and oncologic surgeries, where evidence is currently limited. Comparative studies evaluating the cost-effectiveness and long-term impacts of unimodal versus multimodal prehabilitation interventions are also needed to guide resource allocation in clinical practice. Additionally, research should explore innovative delivery methods, such as telehealth-supported home-based programs, to improve accessibility and adherence among diverse populations. Investigating the role of prehabilitation in vulnerable groups, such as frail elderly patients or those in low-resource settings, could provide further insights into its broader applicability. These studies will help strengthen the evidence base, inform clinical guidelines, and optimize the design of prehabilitation programs to achieve maximal patient benefit.

## Conclusions

This systematic review underscores the transformative potential of prehabilitation in improving postoperative outcomes across diverse surgical systems. The findings demonstrate that multimodal prehabilitation programs, which integrate exercise, nutrition, and psychological support, can significantly reduce complications, enhance functional recovery, and decrease hospital LoS. These benefits emphasize the critical need for standardized, evidence-based prehabilitation protocols tailored to specific patient populations and surgical contexts to maximize outcomes. As global surgical demands continue to rise, integrating prehabilitation into routine perioperative care presents an opportunity to enhance patient recovery, reduce healthcare resource burdens, and drive further advancements in perioperative medicine. Establishing prehabilitation as a core component of surgical planning could pave the way for more efficient, patient-centered care pathways in both high-resource and low-resource settings.
